# Genomic Sequence of Saccharomyces cerevisiae BAW-6, a Yeast Strain Optimal for Brewing Barley Shochu

**DOI:** 10.1128/genomeA.00228-18

**Published:** 2018-04-05

**Authors:** Yasuhiro Kajiwara, Kazuki Mori, Kosuke Tashiro, Yujiro Higuchi, Kaoru Takegawa, Hideharu Takashita

**Affiliations:** a Research Laboratory, Sanwa Shurui Co., Ltd., Oita, Japan; b Computational Bio-Big Data Open Innovation Laboratory, National Institute of Advanced Industrial Science and Technology, Tokyo, Japan; c Department of Bioscience and Biotechnology, Faculty of Agriculture, Kyushu University, Fukuoka, Japan

## Abstract

Here, we report the draft genome sequence of Saccharomyces cerevisiae strain BAW-6, which is used for the production of barley shochu, a traditional Japanese spirit. This genomic information can be used to elucidate the genetic basis underlying the high alcohol production capacity and citric acid tolerance of shochu yeast.

## GENOME ANNOUNCEMENT

Shochu, a traditional Japanese distilled spirit, is produced from various ingredients, including rice, barley, sweet potato, buckwheat, and crude cane sugar (1). The production of shochu is usually carried out by simultaneous saccharification and fermentation in the presence of citric acid, which is produced by koji mold (Aspergillus luchuensis or A. luchuensis mut. kawachii) (2, 3). BAW-6 (previously named W-6) is a Saccharomyces cerevisiae strain that is suitable for the production of barley shochu (4). It was isolated following repeated fermentation of barley shochu mash using S. cerevisiae Kagoshima no. 2. Specifically, selective pressure to survive in higher concentrations of citrate and alcohol during repeat fermentation led to the emergence of naturally occurring Kagoshima no. 2 mutants (4, 5). Here, we conducted genome-wide sequencing to identify candidate mutations that confer the BAW-6 strain with the ability to efficiently produce shochu.

Genomic DNA from BAW-6 was subjected to long-read sequencing by PacBio RS II and short-read sequencing by using the MiSeq platform (Illumina). The reads from both platforms were combined to obtain a final genome sequence. PacBio read sequences were assembled with Canu version 1.5. The original reads were mapped to contigs with pbalign version 0.3.1, and errors were corrected using GenomicConsensus version 2.1. MiSeq reads were mapped using Burrows-Wheeler Aligner (BWA) version 0.7.12, and errors were corrected using Pilon version 1.22. Prediction of coding regions on chromosomes was performed using Augustus version 3.2.3, with S. cerevisiae S288C as a gene model, and Exonerate version 2.4.0 was used for mitochondrial gene prediction (mapping of known gene amino acid sequences). Functional annotations of genes were mapped to genes registered in the Saccharomyces Genome Database using BLAST. A draft genome was thus derived from 37 contigs (36 generated from chromosomes and 1 from mitochondrial DNA). The total base length of the contigs was 11,872,199 bp, and the GC content was 38.3%. The estimated number of genes in the chromosome was 5,425.

Because BAW-6 was derived from the Kagoshima no. 2 strain, the similarity of the two genomes was predicted. Watanabe et al. reported that dysfunction of the Rim15 protein caused by a stop mutation in the C-terminal region of the *RIM15* gene conferred high alcohol-producing properties on sake yeast strain K7 (6). Similarly, Mori et al. reported that a stop mutation exists in the *RIM15* gene of Kagoshima no. 2 (7). However, this stop mutation was not found in BAW-6. Thus, we conclude that the enhanced alcohol production observed in BAW-6 is likely due to alterations in other coding regions. Furthermore, we found that BAW-6 produces more indole compounds than other yeasts that are used to make barley shochu mash. Since indole has an influence on the taste of shochu (8), we suggest that further examination of the BAW-6 genome will assist in the delineation of factors that regulate the concentration of aromatics that are important for the taste of alcoholic beverages.

### Accession number(s).

The draft genome sequences for Saccharomyces cerevisiae strain BAW-6 have been deposited in DDBJ/GenBank under accession numbers BFAW01000001 to BFAW01000037.
